# *Helicobacter pylori vacA* Genotypes in Chronic Gastritis and Gastric Carcinoma Patients from Macau, China

**DOI:** 10.3390/toxins8050142

**Published:** 2016-05-05

**Authors:** Ines Pinto-Ribeiro, Rui M. Ferreira, Sellma Batalha, Thazin Hlaing, Sio In Wong, Fatima Carneiro, Ceu Figueiredo

**Affiliations:** 1 i3S—Instituto de Investigação e Inovação em Saúde, Universidade do Porto, Porto 4200-135, Portugal; iribeiro@ipatimup.pt (I.P.-R.); ruif@ipatimup.pt (R.M.F.); fcarneiro@ipatimup.pt (F.C.); 2 Ipatimup—Institute of Molecular Pathology and Immunology of the University of Porto, Porto 4200-135, Portugal; 3 Centro Hospitalar Conde São Januário, Macau, China; batalhacosi1210@yahoo.co.uk (S.B.); thazin@macau.ctm.net (T.H.); siwong2004@ssm.gov.mo (S.I.W.); 4 Department of Pathology and Oncology, Faculty of Medicine of the University of Porto, Porto 4200-319, Portugal; 5 Department of Pathology, Centro Hospitalar São João, Porto 4200-319, Portugal

**Keywords:** *Helicobacter pylori*, *vacA* gene, genotyping, gastric carcinoma, China

## Abstract

*Helicobacter pylori* is the major triggering factor for gastric carcinoma, but only a small proportion of infected patients develop this disease. Differences in virulence observed among *H. pylori* strains, namely in the vacuolating cytotoxin *vacA* gene, may contribute to this discrepancy. Infection with *vacA* s1, i1 and m1 strains increases the risk for progression of gastric premalignant lesions and for gastric carcinoma. However, in East Asian countries most of the *H. pylori* strains are *vacA* s1, regardless of the patients’ clinical status, and the significance of the *vacA* i1 and m1 genotypes for gastric carcinoma in this geographic area remains to be fully elucidated. The aim of the present study was to investigate this relationship in 290 patients from Macau, China. Using very sensitive and accurate genotyping methods, we detected infection with *vacA* i1 and with *vacA* m1 strains in, respectively, 85.2% and 52.6% of the patients that were infected with single genotypes. The prevalence of *cagA*-positive strains was 87.5%. No significant associations were observed between *vacA* genotypes or *cagA* and gastric carcinoma. It is worth noting that 37.5% of the infected patients had coexistence of *H. pylori* strains with different *vacA* genotypes. Additional studies directed to other *H. pylori* virulence factors should be performed to identify high risk patients in East Asia.

## 1. Introduction

Gastric carcinoma is the fifth most incident and the third most common cause of cancer-related death worldwide. Half of the cases have occurred in East Asian countries, mainly in China, which has the highest estimated mortality rates (24 per 100,000 in men, 9.8 per 100,000 in women) [1].

The major risk factor for the development of gastric carcinoma is persistent *Helicobacter pylori* infection [2]. *H. pylori* induces chronic superficial gastritis that may evolve to chronic atrophic gastritis, intestinal metaplasia, dysplasia and finally to gastric carcinoma [3]. Only a small number of infected individuals will progress towards carcinoma, and it has been shown that disease development depends on the interplay between host genetic susceptibility, environmental factors, and bacterial virulence factors [4,5,6].

*H. pylori* strains are genetically very heterogeneous and genetic variation within genes encoding virulence factors contributes to differences in the pathogenicity of strains [7]. One of the most studied *H. pylori* virulence-associated genes is *cagA*, encoding the CagA effector protein that is translocated into the host cells by a type IV secretion system [8,9]. In Western countries, approximately 70% of the *H. pylori* strains contain the *cagA* gene, and infection with this type of strain has been associated with increased risk for gastric atrophy, intestinal metaplasia and gastric carcinoma [4,10,11]. In East Asian countries, however, the prevalence of *cagA*-positive strains is higher than 90%, making it difficult to disclose its impact in gastric carcinoma [12,13].

The *vacA* gene, encoding the vacuolating cytotoxin A (VacA), is another *H. pylori* virulence-associated gene that exhibits variation among strains [14]. Although *vacA* is present in all *H. pylori* strains, it shows allelic variation in three main regions: the signal (s)-region that contains the s1 (s1a, s1b, and s1c) or s2 alleles, the intermediate (i)-region that contains the i1 or i2 alleles, and the middle (m)-region that contains the m1 or m2 (m2a and m2b) alleles [15,16,17]. Different combinations of *vacA* s, i and m alleles may occur, resulting in VacA toxins with distinct abilities to induce vacuolation in epithelial cells [15]. It has been shown that while *vacA* s1/m1 strains are vacuolating and *vacA* s2/m2 are non-vacuolating, the *vacA* s1/m2 strains may or not induce vacuolation depending on the i-region genotype. Therefore, *vacA* s1/m2 strains that contain the i1 allele are vacuolating, whereas strains that contain the i2 allele are non-vacuolating [15,16,18]. Numerous studies have shown that in Western countries, *H. pylori vacA* s1 and m1 strains are associated with enhanced gastric mucosal inflammation and with increased risk for atrophy, intestinal metaplasia and carcinoma in comparison with *vacA* s2 and m2 strains [11,19,20]. Additional studies including patients from Western countries also found associations between infection with strains containing the *vacA* i1 allele and increased risk for progression of gastric precancerous lesions and for gastric carcinoma [18,21,22,23].

In East Asian countries, however, the relationship between *H. pylori vacA* s-region genotypes and gastric carcinoma has been difficult to ascertain, as virtually all strains are *vacA* s1 [12,13]. In contrast, the *vacA* m-region appears to be more variable among East Asian strains, with the m1 allele predominating in Japan and South Korea, and the m2 allele predominating in Southeast Asia, including countries such as Vietnam [24]. Few studies have evaluated the clinical significance of the *vacA* i region in gastric carcinoma in East Asia [25,26], namely in China. Therefore, we investigated the relationship between *H. pylori vacA* i- and m-region genotypes and gastric carcinoma development in patients living in Macau, China.

## 2. Results

### 2.1. H. pylori vacA Genotypes and cagA Status

Two hundred and eighty-one (96.9%) from a total of 290 patients included in the study were positive for *H. pylori* as shown by PCR.

The characterization of the *vacA* i-region was obtained in 272 (96.8%) of *H. pylori*-infected patients, in which 236 (86.8%) patients were infected with a single strain, while the remaining 36 (13.2%) were infected with multiple *H. pylori* strains. Of the patients with single infections, 201 (85.2%) were infected with *vacA* i1 strains and the remaining 35 (14.8%) patients were infected with i2 strains (Table 1).

Characterization of the *vacA* m-region was successful in all but one case. Two hundred and eleven (75.4%) samples contained a single allele: 111 (52.6%) were genotyped as m1 and 100 (47.4%) as m2 (Table 1); 93.0% of the m2 strains were of the m2a subtype, and the remaining 7.0% of strains were m2b. In 69 (24.6%) patients, more than one m allele was detected, and among these the m1/m2a combination was the most frequent (*n* = 65, 94.2%).

Approximately 95% of *H. pylori*-infected patients were successfully characterized for the *vacA* s-region. Multiple *vacA* s-region genotypes were observed in 27 (9.8%) patients, and within single infections the *vacA* s1 allele was detected in 227 (91.2%) cases and the s2 allele in 22 (8.8%) cases (Table 1). Most of the *vacA* s1 strains were of the s1c subtype (71.4%), while the s1a and the s1b alleles were detected in 33 (14.5%) and eight (3.5%) strains, respectively. In 24 (10.6%) samples, two *vacA* s subtypes were simultaneously detected: s1a/s1b (*n* = 4), s1a/s1c (*n* = 17), and s1b/s1c (*n* = 3), and these samples were considered as *vacA* s1 for the remaining analyses. Among 27 patients infected with multiple strains, the following combinations of s alleles were found: s1a/s2 (*n* = 6; 22.2%), s1c/s2 (*n* = 11; 40.8%); s1a/s1b/s2 (*n* = 2; 7.4%); and s1a/s1c/s2 (*n* = 8; 29.6%).

Considering all *vacA* regions together, the following genotype combinations were observed: s1/i1/m1 (*n* = 71, 41.8%), s1/i1/m2 (*n* = 67, 39.4%), s1/i2/m1 (*n* = 9, 5.3%), s1/i2/m2 (*n* = 7, 4.1%), s2/i1/m1 (*n* = 5, 2.9%), s2/i2/m1(*n* = 4, 2.4%), s2/i1/m2 (*n* = 4, 2.4%), and s2/i2/m2 (*n* = 3, 1.8%). It is noteworthy that a total of 102 (37.5%) patients were infected with multiple strains (Table 1).

The prevalence of *cagA*-positive strains was 87.5% (Table 1). Combining the *vacA* genotypes with the *cagA* status, 13 different genotypic combinations were found. The most prevalent ones were *vacA* s1/i1/m1/*cagA*-positive (*n* = 67, 39.4%) and *vacA* s1/i1/m2/*cagA*-positive (*n* = 64, 37.6%). The *vacA* s2/i2/m1/*cagA*-negative genotype was the least prevalent (*n* = 1, 0.6%), and *vacA* s2/i2/m2/*cagA*-negative strains were not found.

### 2.2. Relationship between vacA i-Region Genotypes, s- and m-Region Genotypes and cagA Status

Regarding the relationship between *vacA* genotypes and *cagA*, *vacA* i1 strains were frequently s1 (89.6%) and *cagA*-positive (90.9%), while *vacA* i2 strains were more commonly s2 (43.8%) and *cagA*-negative (56.2%; *p* = 0.002 and *p* < 0.001, respectively; Table 2). The prevalence of the i1 allele was similarly detected among m1 (85.4%) and m2 (87.7%) strains, and no relationship was observed between the genotypes of these *vacA* regions (Table 2).

### 2.3. Relationship between H. pylori Genotypes and Gastric Carcinoma

The 281 *H. pylori*-infected patients comprised 234 patients with chronic gastritis (mean age 58.0 ± 15.1 years; female:male ratio of 1.13:1) and 47 with gastric carcinoma (mean age 66.6 ± 14.1 years; male:female ratio of 2.4:1).

*H. pylori vacA* i1, s1, and m1, as well as *cagA*-positive strains, were found with similar frequencies in patients with chronic gastritis and patients with gastric carcinoma, and consequently, no significant relationships could be disclosed between *vacA* or *cagA* genotypes and gastric carcinoma (Table 1). Also, no significant differences were observed between *vacA* or *cagA* genotypes and degree of atrophy, presence of intestinal metaplasia, or dysplasia. Overall, 37.5% of the patients were infected with multiple strains as assessed by *vacA* genotyping. No relationship was observed between infection with single/multiple *H. pylori* strains and gastric carcinoma (Table 3).

## 3. Discussion

Gastric carcinoma is one of the most incident and deadly cancers worldwide. The incidence of gastric carcinoma is particularly high in East Asian countries, where the prevalence of *H. pylori* infection is also high [1,2,27,28]. *H. pylori* strain variation, especially that related to *vacA* and *cagA* virulence factor-encoding genes, has been proposed as a means to identify strains with the highest degrees of pathogenicity and, consequently, individuals with the highest risk of disease [29].

This study constitutes the largest case-control study in East Asia to address *H. pylori* virulence factor genotyping and gastric carcinoma. Here, we have characterized the *H. pylori vacA* i- and m-regions in Chinese patients with chronic gastritis and with gastric carcinoma from Macau. In the *vacA* i-region, the i1 allele was dominant and no differences in allelic prevalence were observed between chronic gastritis and gastric carcinoma patients. These results contrast with those of earlier studies in Iranian, Italian, Portuguese, Spanish, Belgian, and UK populations, where the *vacA* i1 allele has been associated with increased risk for atrophic gastritis, intestinal metaplasia, and gastric carcinoma [16,18,21,22,23,30]. However, our results in the Chinese population of Macau are consistent with those of studies that include patients from East Asian countries, namely Japan, South Korea, and China, and that report a prevalence of over 95% of the *vacA* i1 allele [25,26,31,32]. Therefore, although the *vacA* i-region genotyping is useful to identify patients at high risk of gastric carcinoma in Western countries, it may have limitations in East Asian countries.

We have observed a similar frequency distribution of the two *vacA* m-region alleles, with 52.6% of the strains in Macau being m1 and the remaining 47.4% being m2. Nevertheless, the frequency distribution of these alleles was similar in chronic gastritis and gastric carcinoma patients, and therefore no relationship between *vacA* m-region genotypes and gastric carcinoma could be established. It has been shown that the m-region genotypes have regional geographic variation between East Asian countries and it has been suggested that the m-region may play a role in the regional differences in gastric carcinoma incidence [24]. The patients from Japan and South Korea are mainly infected with *vacA* m1 strains [12,26], while m2 strains are more prevalent in patients from Vietnam or Hong Kong [33,34], possibly reflecting the higher incidence of gastric carcinoma in the former than in the latter. Within mainland China, a significant geographic diversity of the *vacA* m-region has also been also described, and the m1 allele frequency was higher in Guangxi, an area with a high gastric carcinoma incidence, than in Beijing [13]. Although these data are in favor of *vacA* m1 strains being more virulent than m2 strains, based on our data, the *vacA* m-region genotypes do not appear to be useful for identifying high-risk individuals in Macau.

Similarly to what was found in the *vacA* i-region, in the s-region the great majority of the strains in Macau contained the s1 allele, with s1c being the most prevalent subtype. Likewise, a very high frequency of *cagA*-positive strains was detected in this population. These observations concur with previous descriptions from Chinese, Korean, and Japanese populations showing a strong predominance of *vacA* s1 and *cagA*-positive strains [12,13,25,35,36]. Due to the high frequency of *vacA* s1 and *cagA*-positive strains in Macau, and in contrast with other geographic areas, including Western, Middle Eastern, Latin American, and African countries [19,20,37,38,39], no relationships with disease outcome could be disclosed.

While the great majority of *H. pylori* strains presented the most virulent genotypic combination of *vacA* s1/i1/*cagA*-positive, the less virulent *vacA* s2/i2/m2/*cagA*-negative strains were not found. An association between the *vacA* i1 and s1 genotypes and between the latter and the presence of *cagA* was observed in this study. The associations between these *vacA* loci and *cagA* have been reported both in Western and in other East Asian populations [18,21,26,31]. The reasons underlying these associations are not very clear. The *vacA* and *cagA* genes are located distantly in the chromosome, and therefore genotype association cannot be due to clonality, nor to genetic linkage, as *H. pylori* is highly recombinatorial [40,41]. Interestingly, it has been proposed that VacA and CagA interplay with each other with the purpose of regulating excessive damage promoted by each of the virulence factors individually, which would probably destroy the *H. pylori* niche [42].

The genotyping of *vacA* directly in gastric biopsy specimens allowed us to identify a high number of patients (37.5%) with coexisting strains with different *vacA* types, which were considered as multiple *H. pylori* infections. The prevalence of multiple infections has now been reported for numerous geographic locations [13,19,43,44,45,46], and in general it appears to be higher in countries where the risk for *H. pylori* infection is also high [47]. Whether the coexistence of strains with different *vacA* genotypes reflects the high prevalence of *H. pylori* in Macau or genotype adaptation to specific niches within a gastric biopsy remains to be disclosed. Nevertheless, the natural history and consequences of the acquisition of the infections with two (or more) strains requires further studies, as there may be clinical/therapeutic implications.

In conclusion, the great majority of the *H. pylori* strains found in Macau were *vacA* s1/i1/*cagA*-positive, independent of the clinical outcome of the infection. Therefore, these factors cannot be used for identifying high-risk patients in this geographic region. Other regions of variation in *vacA,* such as the d-region [25], in other *H. pylori* virulence-associated factors, namely in the *cagA* gene promoter [48,49] or EPIYA-encoding region [36,50], may represent alternative markers of *H. pylori* virulence in East Asian populations.

## 4. Materials and Methods

### 4.1. Patients and Study Population

A total of 290 paraffin-embedded gastric tissue specimens from patients living in Macau were characterized for *H. pylori vacA* genotypes and for the presence of the *cagA* gene. Samples included 238 cases of chronic gastritis (mean age 60.8 ± 14.2 years; female:male ratio of 1.1:1), comprising patients with superficial gastritis (*n* = 82) and atrophic gastritis (*n* = 156); and 52 cases of gastric carcinoma (mean age 66.6 ± 13.6 years; male:female ratio of 2.2:1). Within the atrophic gastritis group, 18 patients had dysplastic lesions (*n* = 15 had mild dysplasia and *n* = 3 had moderate dysplasia). Evidence of epithelial erosions/ulcerations was present in 79 cases with chronic gastritis. Patients were selected consecutively from the registry of Centro Hospitalar Conde de São Januário (CHCSJ), Macau, China. The study population was composed by Chinese (*n* = 282; 97.2%), Macanese (*n* = 6; 2.1%), and Asian (*n* = 2; 0.7%) living in Macau. Samples were delinked and unidentified from their donors. The study was approved by the institutional review board of CHCSJ, registry of entry no. 5875, approved on 18 August 2008.

### 4.2. Histopathology

Two gastric biopsy specimens from the antrum (one from the greater curvature and one from the incisura angularis) and one from the corpus were immersed in 10% formalin and embedded in paraffin. Sections were stained with hematoxylin and eosin, Alcian blue-periodic acid-Schiff, and modified Giemsa. Only cases with adequate sized biopsy specimens of both antral and corpus mucosa were accepted for histological assessment by an experienced pathologist, who was blinded with respect to the clinical information of each patient. The histopathological parameters were classified according to the criteria described in the updated Sydney’s classification system [51]. Gastric carcinoma cases were categorized according to Lauren’s and Carneiro’s classification [52,53].

### 4.3. DNA Isolation

*H. pylori* detection and characterization was performed in DNA extracted from paraffin-embedded gastric tissue. Cuts of 10 μm of formalin-fixed and paraffin-embedded blocks were obtained from each biopsy specimen, changing the blade after each block to avoid cross-contamination. Total DNA was isolated after digestion in a solution containing 10 mM Tris-HCl (pH 8.0), 5 mM EDTA, 0.1% sodium dodecyl sulphate (SDS), and 0.1 mg/mL proteinase K for at least 12 h at 55 °C. Proteinase K was inactivated by incubation for 10 min at 95 °C.

DNA isolation from *H. pylori* strains 60190 (ATCC 49503; *vacA* s1/i1/m1 and *cagA*-positive) and Tx30a (ATCC 51932; *vacA* s2/i2/m2 and *cagA*-negative) was performed using the GRS Genomic DNA kit-Bacteria (GRISP, Porto, Portugal), following the manufacturer’s instructions. DNAs from these strains were used as controls for genotyping.

### 4.4. H. pylori vacA Genotyping and cagA Gene Detection

*H. pylori vacA* i-region was genotyped by allele-specific polymerase chain reaction (PCR) using the forward primer VacIABF and the reverse primers C1R, and C2R, as previously reported [16,23]. PCR mixtures were prepared in a volume of 25 µL, containing 1x PCR buffer (Applied Biosystems, Foster City, CA, USA), 2.5 mM MgCl_2_ (Applied Biosystems), 1 mM concentrations of deoxynucleotide triphosphates (dNTP, Nzytech, Lisbon, Portugal), 0.5 U of AmpliTaq Gold (Applied Biosystems), and 0.5 µM concentrations of forward and reverse primers. PCR was performed with a 9 min predenaturation at 95 °C, followed by 45 cycles of 30 s at 95 °C, 45 s at 50 °C, and 45 s at 72 °C. Final extension was performed for 10 min at 72 °C. PCR of the human glyceraldehyde3-phosphate dehydrogenase (GAPDH) housekeeping gene was used to monitor the DNA isolation procedure. PCR products were electrophoresed on 2% agarose gels and examined under UV light, according to standard procedures. Genotyping of *vacA* s- and m-regions and detection of *cagA* were performed by PCR followed by reverse hybridization on a line probe assay (LiPA), as previously described [19,54]. Patients were considered to be infected with multiple *H. pylori* strains, unless otherwise stated, when *vacA* typing detected more than one allele at each of the s-, i-, or m-regions.

### 4.5. Statistical Analyses

Associations between *vacA* genotypes and *cagA*, and the relationships between genotypes and disease were assessed using the Fisher’s exact test. Age and gender frequencies between groups of patients were compared with Student’s *t-*test and with Fisher’s exact test, respectively.

Statistical analyses were computed with the software Statview for Windows (Version 5; SAS Institute, Cary, USA, 1998). Differences were considered to be statistically significant at *p* values < 0.05.

## Figures and Tables

**Table 1:** *H. pylori vacA* genotypes and *cagA* status in patients from Macau, China.

	Chronic Gastritis (*n* = 234)	Gastric Carcinoma (*n* = 47)	*p*-Value	Total
***vacA* i-region ^1^**				
**i1**	172 (85.1%)	29 (85.3%)	>0.999	201 (85.2%)
**i2**	30 (14.9%)	5 (14.7%)		35 (14.8%)
***vacA* m-region ^2^**				
**m1**	94 (53.7%)	17 (47.2%)	0.583	111 (52.6%)
**m2**	81 (46.3%)	19 (52.8%)		100 (47.4%)
***vacA* s-region ^3^**				
**s1**	189 (91.7%)	38 (88.4%)	0.553	227 (91.2%)
**s2**	17 (8.3%)	5 (11.6%)		22 (8.8%)
***cagA* status**				
**positive**	208 (88.9%)	38 (80.9%)	0.146	246 (87.5%)
**negative**	26 (11.1%)	9 (19.1%)		35 (12.5%)

^1^ Nine cases (three gastritis and six carcinomas) could not be genotyped and multiple strains were detected in 36 cases (29 gastritis and seven carcinomas); ^2^ One carcinoma case could not be genotyped and multiple strains were detected in 69 cases (59 gastritis and 10 carcinomas); ^3^ Five cases of gastritis could not be genotyped and multiple strains were detected in 27 cases (23 gastritis and four carcinomas).

**Table 2:** Relationship between *vacA* i-region genotypes with s- and m-region genotypes, and with *cagA* status.

*vacA* i-Region	*vacA* s-Region		*vacA* m-Region		*cagA* Status	
	s1	s2	m1	m2	Positive	Negative
***vacA* i1**	138 (89.6%)	9 (56.3%)	76 (85.4%)	71 (87.7%)	140 (90.9%)	7 (43.8%)
***vacA* i2**	16 (10.4%)	7 (43.8%)	13 (14.6%)	10 (12.3%)	14 (9.1%)	9 (56.2%)
***p*-value**	0.002		0.823		<0.001	

Only samples completely genotyped and with single genotypes for the *vacA* s-, m- and i-regions are included.

**Table 3:** Relationship between single and multiple *H. pylori* infections and gastric carcinoma.

*H. pylori*	Chronic Gastritis	Gastric Carcinoma	*p*-Value	Total
**Single infections ^1^**	142 (62.0%)	28 (65.1%)	0.735	170 (62.5%)
**Multiple infections ^2^**	87 (38.0%)	15 (34.9%)		102 (37.5%)

^1^ Nine samples could not be genotyped; ^2^ As evaluated by the presence of more than one allele at each of the *vacA* s-, i-, or m-regions.
